# The value proposition of efficiency discount options: The government employees medical scheme emerald value option case study

**DOI:** 10.4102/phcfm.v13i1.2292

**Published:** 2021-01-20

**Authors:** Michael M. Willie, Barry Childs, Gunvant Goolab

**Affiliations:** 1 Policy Research and Monitoring, Council for Medical Schemes, Pretoria, South Africa; 2 Insight Actuaries and Consultants, Actuarial Society of South Africa, Cape Town, South Africa; 3 Government Employees Medical Scheme, Pretoria, South Africa

**Keywords:** efficiency discount options, cost of care, medical schemes, Emerald Value Option, Government Employees Medical Scheme, general practitioner consultations, specialist consultations

## Abstract

**Background:**

The Government Employees Medical Scheme (GEMS) introduced an EDO named the Emerald Value Option (EVO) in January 2017. The option was introduced to contain the cost of care whilst simultaneously improving the quality of care by championing care coordination.

**Aim:**

This study aimed to assess the impact of introducing an EDO such as EVO as a cost-containment strategy using contracted provider networks and coordinated care.

**Setting:**

The study was conducted using aggregated data from GEMS. Government Employees Medical Scheme is a restricted medical scheme available to government employees in South Africa.

**Methods:**

This is a descriptive pairwise comparison study between the Emerald benefit option (the parent option), which does not have embedded care coordination, and its derivative, EVO.

**Results:**

Membership and claims data for 2018 were analysed. Expenditure per life per month in 2018 on the EVO amounts to R1357.01. After adjusting for the risk profile of beneficiaries on the EVO, expenditure per life per month would be expected to be R1621.73 (based on the conventional Emerald option). This translates to a savings of 16.3%. Similarly, health outcomes for EVO were more favourable than expected, actual admission rates were lower at 23.2% versus 26.2% expected.

**Conclusions:**

The EVO benefit design has succeeded in lowering the cost of care through network provider contracting and care coordination. The EVO has saved approximately R490 million in healthcare costs in 2018. If applied across the medical schemes industry, it is estimated that EVO contracting, and care coordination principles could save R20 billion per annum.

## Introduction

### Environment

Medical schemes in South Africa operate under the Medical Scheme’s Act 131 of 1998. Schemes operate as not-for-profit entities under a set of solidary-based principles, including open enrolment, community rating and the provision of prescribed minimum benefits across all benefit options. There are two types of medical schemes – open medical schemes, which are open to accepting membership from any applicant, and restricted medical schemes, which are open only to a specific employer or industry or other affiliation. Approximately 16% of the South African population are members of medical schemes.

The Government Employees Medical Scheme (GEMS) is a restricted medical scheme available only to government employees. It is the largest restricted medical scheme in South Africa, making up almost 20% of the medical scheme population. It has five benefit options and one efficiency discount option (EDO) that offer a range of available benefits and limits, in return for income-based contributions.

### Legislative requirements

Efficiency discount options in medical schemes operate as a special dispensation whereby schemes offer discounts on monthly contributions by managing access to care to directly contracted networks of healthcare providers.^1^ Such options are subject to an exemption approval process by the Council for Medical Schemes (CMS). Efficiency discount option benefit options are treated as derivative options and offer the same benefits and limits as their respective ‘parent’ option. In addition to making use of a network of providers, Emerald Value Option (EVO) incorporates general practitioner (GP) nomination, GP to specialist referral and care coordination, which was approved by the CMS in its exemption process.

### Literature review

Care coordination relates to the relationships and referral patterns between GPs and specialists.^2^ It promotes a better relationship between the patient and providers such that relationships are optimally managed. One of the key principles is the emphasis on the primary healthcare provider as the first point of entry when seeking care.^3,4^ Initially, the primary reason for introducing the principle of the referral or ‘the gatekeeping role’, was the protection of the income of GPs; however, it has proved to be a sensible and important way of regulating and coordinating primary and secondary medical care.^5^ Other studies depict the role primary healthcare providers play in containing costs. A study by Willie and Gantsho^6^ discussed the GP as a proxy for accessing chronic benefits and the role of primary healthcare within the managed care environment as an effective tool to avoid costly hospitalisation. Other studies by McWilliams^7^ also suggest that care coordination can improve clinical outcomes while lowering costs.

A case study to tangibly compare the effects of a gatekeeper model and an open-access model has been conducted on the management of patients with chest pain. Patients of similar demographic profiles with chest pain who were referred to a cardiologist were studied, of which 490 were referred from a gatekeeper model and 924 referred from an open-access model. It was found that open-access patients were twice as likely to be referred to a cardiologist, resulting in higher total cardiologist fees.^8^ This implies that in an open-access model, patients see specialists more freely, even if it may not be necessary, which increases costs for medical schemes. The study showed cardiologist fees per patient were lower in the gatekeeper model group ($972 on average) than the open-access group ($1187 on average).

One study showed that European countries with a GP referral system spent 7.8% of their Gross National Product (GNP) on healthcare in comparison to European countries without this system, which spent 8.6% of their GNP.^9^

Another consideration when looking at specialist referral rates is the method by which GPs are remunerated. A Canadian study showed that a fee-for-service model resulted in the lowest referral rate followed by an interdisciplinary capitation model and then a non-interdisciplinary capitation model. It is expected that a fee-for-service model will result in lower referrals than a capitation model because of the reduced compensation for services delivered by GPs with a capitation model. Furthermore, it is expected that an interdisciplinary capitation model will have lower referral rates because interdisciplinary practices have more resources and chances for specialists and physicians to work together.^10^

The above study links higher levels of care coordination, prevalent in multidisciplinary teams, to lower levels of specialist referrals. Clarke et al.^11^ argues that there is a major weakness in typical outpatient and inpatient care delivery systems. The author furthered depicts the silo effect of primary care professionals, paramedics, emergency physicians, and hospitalists function and how these impede care coordination, inhibit communication, compromise quality, and raise costs.

A German study compared patients in a gatekeeping model and patients in a control group to see how they differed with respect to care coordination. The study found that patients in the gatekeeping model were more likely to enrol in disease management programmes, which implies a higher level of coordinated care. The study showed that along with a better care coordination, the gatekeeping group also prevented hospitalisation to a larger extent than the control group.^12^

The National Health Insurance (NHI) White Paper issued indicated that the health system is organised into three areas of healthcare service delivery being Primary Health Care Services (PHC), Hospital and Specialised Services and Emergency Medical Services (EMS). Primary Health Care Services is referred to as the heartbeat of the NHI, being the first point of contact with the health system, which is critical to ensure health system sustainability, receiving the care needed at this level or be referred to a hospital if more specialised services are necessary.^13^

The Health Market Inquiry Report indicated that the analysis of the GEMS EDO further highlights the potential for networks to foster competition amongst facilities and thereby result in savings. The evidence available, particularly from EDOs that are able to directly compare the impact following the network adoption, show substantial benefits from networks, including beneficiary growth and improvements in net healthcare results.^14^

### Trends on efficiency discount options

The CMS started reporting on EDOs in 2016. The key trends depicted in the CMS annual reports show that EDOs perform better than their non-discounted comparator options. The CMS reports show that 11 benefit options offered EDO-discounted equivalents in 2017. Just over 20% of the beneficiaries were on EDO benefit options in 2017. The proportion of beneficiaries on non-EDOs was just under 80% in 2017. The CMS report further depicts that the average age of the membership of EDOs is lower than that of the derivative option. As of 31 December 2016, the average EDO member is 31.6 years, which is younger than the average member on the non-EDO, which was 35.2 years. Financial results for EDOs were better than their non-discounted counterparts, with EDO options contributing 63% of the schemes’ operating surpluses while accounting for only 24% of the membership.

Government Employees Medical Scheme experience with EDO through its EVO is unlike the EDO option experience of open schemes. The EVO initially attracted an older age profile and more severe risk profile than its non-discounted benefit option comparator, Emerald. Sicker members appear to have joined the option to make use of the coordinated care model and lower contributions.

**FIGURE 1:**
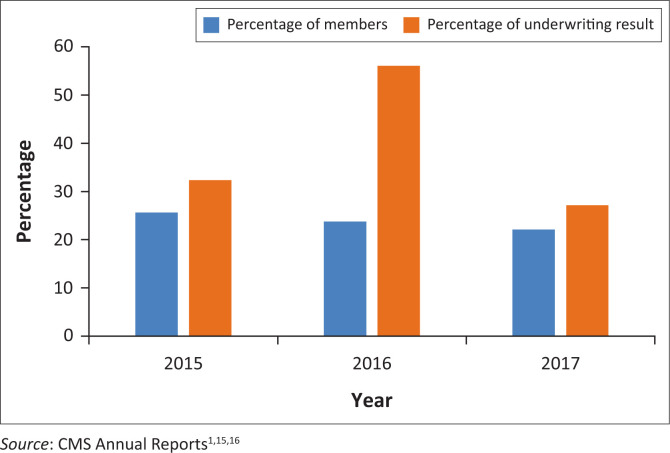
Membership and financial performance of efficiency discount option benefit options.

Beneficiaries on EDO options contribute a high portion of medical scheme underwriting surplus relative to the proportion of beneficiaries, as illustrated in Figure 1.

**FIGURE 2:**
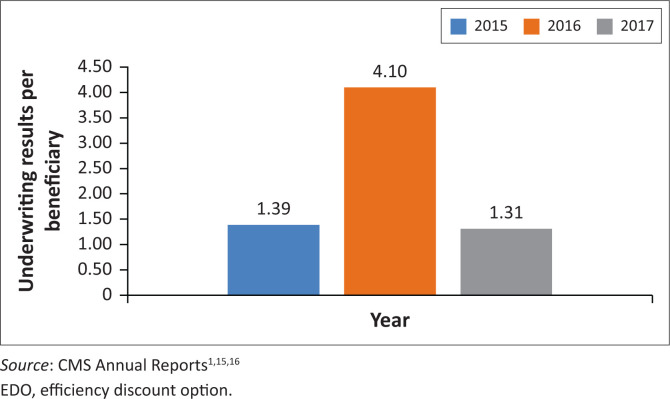
Relative efficiency discount option underwriting results per beneficiary.

Beneficiaries on EDO options make higher surpluses per beneficiary than non-EDO options on the same medical schemes, with the multiple ranging from 1.31 in 2017 to 4.10 multiple over the period 2015 to 2017, as shown in Figure 2.

## Methodology and analysis

### Materials

Beneficiary demographic and claims data were used to compare the claims experience of EVO against the claims experience of the Emerald option.

### Setting

Emerald is GEMS’ largest benefit option, accounting for 1 107 742 beneficiaries in December 2018 (60% of the scheme beneficiaries). Emerald Value Option accounted for 180 594 beneficiaries in December 2018 (10%). The benefits and limits are the same for both options, with EVO funding care at a network of hospitals, requiring beneficiaries to nominate their main GP and further requiring GP referral to gain access to specialist visits.

### Design

This is a descriptive study that compares the costs of care between EVO and its non-discounted comparator option, Emerald. The main objective was to determine the value proposition of EVO (cost, contributions, risk profiles and other attributes, such as claims experience and utilisation). Claims from January 2017 to June 2019 were considered.

Subject to the application of care coordination and the appointed hospital network, the benefits on EVO and the Emerald option are equivalent. Hence, one can directly measure the impact of care coordination by contrasting claims data between the Emerald and Emerald Value benefit options without the interference of benefit design limits. Comparisons are considered after risk adjustment. Risk adjustment is necessary to account for differences in the profile of beneficiaries between the two options which affect claims costs. Risk adjustment factors include age, gender and the number of chronic conditions. Data were aggregated across beneficiaries and were not identifiable.

### Ethical consideration

This article followed all ethical standards for a research without direct contact with human or animal subjects.

### Results

**FIGURE 3:**
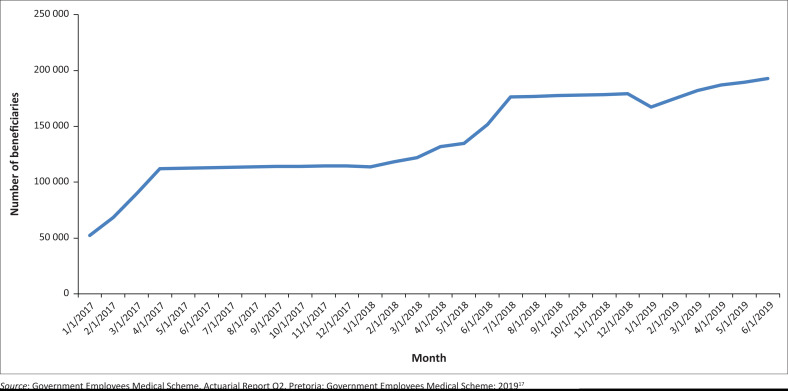
Emerald value option-covered beneficiaries by month.

Since January 2017, the Emerald Value sub-option has grown by more than 40 000 families (over 100 000 individual beneficiaries) as depicted in Figure 3. On average, these families are larger and have a higher pensioner ratio than other Emerald members. Growth continued into 2018, which showed continued support for EVO. Table 1 shows membership and demographic information between the parent option Emerald and the Emerald Value sub-option.^17^ The age profile of the two options was not significantly different, however the Emerald Value option attracted a much younger profile of members compared to the parent option.

**TABLE 1:** Membership June 2019.

Dimension of analysis	Emerald value	Emerald
**Number of principal members**	70 064	425 314
Average age	47.1	48.8
% Male	31.1	26.3
% Chronic users	44.5	46.8
% Over age 60	14.8	16.5
**Number of beneficiaries**	194 389	1 079 000
Beneficiary ratio	2.8	2.5
Average age	31.7	32.7
% Adult beneficiaries	21.7	19.3
% Chronic users	26.7	27.7
% Over age 60	11.0	11.6

*Source*: Government Employees Medical Scheme. Actuarial Report Q2. Pretoria: Government Employees Medical Scheme; 2019^17^

**FIGURE 4:**
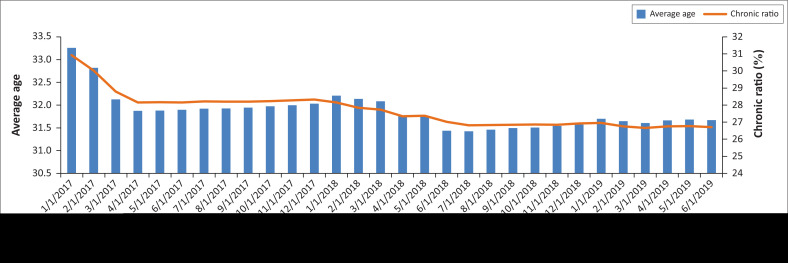
Emerald value option average beneficiary age and chronic ratio.

Figure 4 depicts the trend of the average age and chronic prevalence of the Emerald Value benefit option from January 2017. Since its inception, the average profile of this option has continued to improve monthly. In April 2018, the average age of Emerald Value was almost identical to the Emerald average age.

**FIGURE 5:**
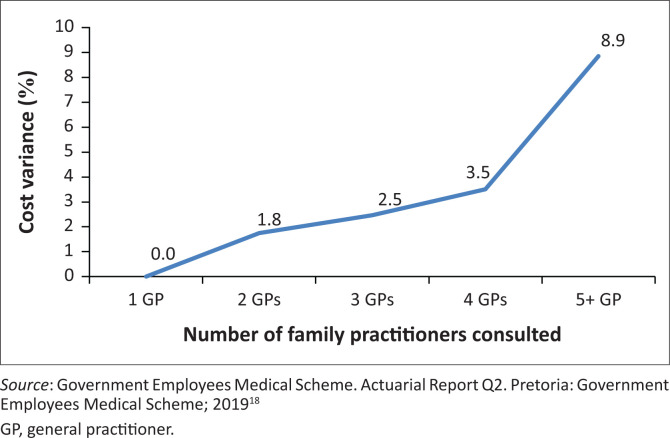
Cost variances associated with the number of family practitioners consulted (benchmarked against using one general practitioner).

In an unconstrained environment, medical scheme beneficiaries frequently go to many different doctors for care, including multiple GPs. Seeing multiple doctors for treatment means fragmented, uncoordinated care, which adds costs to the system. Patients who seek care from one principal doctor incur lower costs than those who seek care from multiple doctors on a risk-adjusted basis. Figure 5 depict cost variance by number of family practitioners consulted.

General practitioner nomination is a process whereby patients select a primary GP who is tasked with coordinating their care and inform GEMS of their selection. Beneficiaries are required to utilise their nominated GP for all consultations, failing which a copayment is applied. Nominated GPs must be part of the GEMS network. Network providers have agreed to bill at scheme tariff and to adhere to predetermined efficiency and quality standards. There are over 6000 GPs on the network across the country. General practitioner nomination is necessary to promote care coordination. The use of a single (nominated) GP helps to create an environment whereby care coordination can naturally propagate as patients look to a single doctor to oversee their care. Nomination helps to cement the relationship between patients and practitioners such that the practitioner is intimately familiar with the patient’s medical history. This enables more effective treatment and limits the potential for duplicative care. Table 2 below depicts a higher proportion of a practitioner nomination compare to more than two practitioners on EVO relative to Emerald.^18^

**TABLE 2:** General practitioner nomination.

Number of GPs	Emerald (%)	EVO (%)
1	74.3	81.3
2	20.6	15.9
3	4.1	2.4
4+	0.9	0.4

*Source*: Government Employees Medical Scheme. Actuarial Report. Pretoria: Government Employees Medical Scheme; 2018^18^

GP, general practitioners; EVO, Emerald value option.

Beneficiaries on the EVO are less inclined to use multiple GPs than beneficiaries on the conventional Emerald option. Proportionally more single (nominated) practitioner were on EVO compared to Emerald as depicted in Table 2. On the conventional Emerald option, 52.4% of the beneficiaries have consulted with a single practitioner. On the EVO, 63.2% of the beneficiaries have consulted with a single practitioner.

As with most medical scheme options on the market in South Africa, access to specialists is not constrained. The Emerald option offers specialist benefits without the need for a referral from a GP. In 2018 on the Emerald option 62% of specialist consultations arose without a preceding GP engagement. On EVO beneficiaries require a referral from a family practitioner before consulting with a specialist. This is necessary to limit unnecessary specialist consultations and to ensure that patients receive medical care at the most appropriate level. In turn, family practitioner to specialist referrals allow for the efficient use of clinical and financial resources.

Beneficiaries who consult with specialists and not GPs have risk-adjusted costs per life per month which are 9.4% higher than the average.

Patients who seek care from specialists without preceding engagements with GPs are even more costly, on a risk-adjusted basis, for GEMS than patients whose doctor hop across more than five GPs.

**FIGURE 6:**
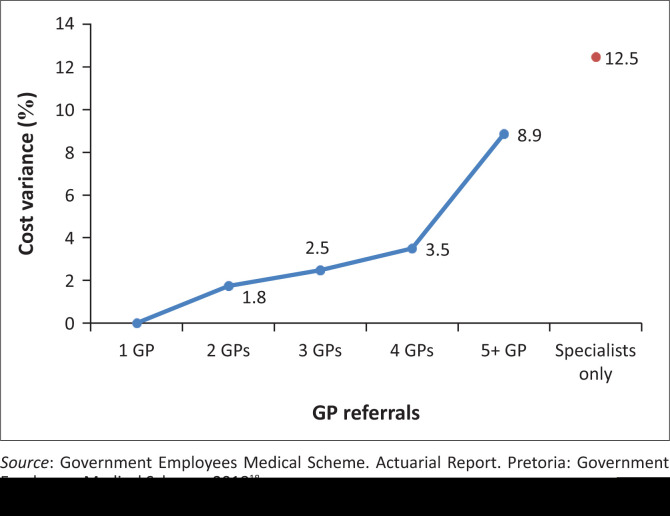
Cost variances for specialist visits without a general practitioner referral.

**FIGURE 7:**
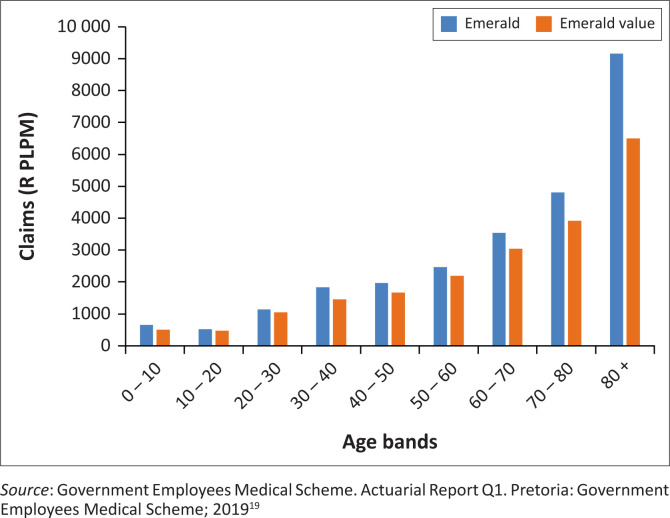
Claims per life per month (PLPM) on EVO and Emerald (2018) by age bands.

**FIGURE 8:**
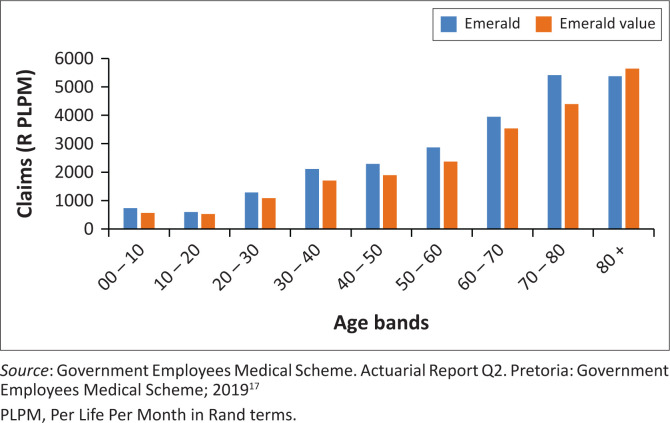
Claims per life per month on EVO and Emerald (Q2 2019) by age bands, (R PLPM) Per Life Per Month in rand terms.

**FIGURE 9:**
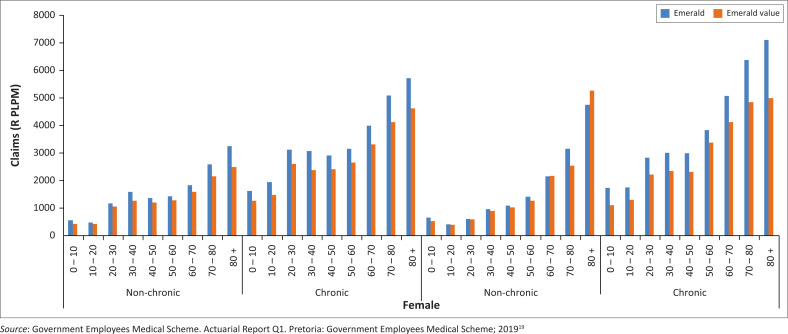
Claims per beneficiary per month on Emerald value option and Emerald by age, gender and chronic status (2018).

**FIGURE 10:**
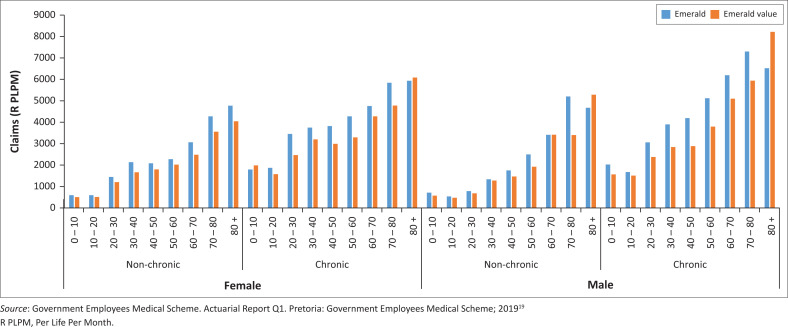
Claims per beneficiary per month on Emerald value option and Emerald by age, gender and chronic status (January 2019 to June 2019).

Figure 6–10 above risk adjusted claims data. Looking at overall costs, when comparing Emerald and EVO claims per beneficiary per month by age group we observe that claims are lower on EVO across age groups.

The lower EVO claims expenditure is further expanded when adjusting for the combination of age, gender and chronic status of the beneficiaries participating in these options. After removing the effect of these demographic differences, we calculate EVO claims to be 17.5% lower than expected when compared to Emerald’s beneficiary risk profile and claims expenditure levels.

Claims expenditure per life per month on the EVO amounts to R1405.24. After adjusting for the risk profile of beneficiaries on the EVO, claims expenditure per life per month would be expected to be R1679.91. The expected figure uses claims expenditure experience from the Emerald option across age group, gender and chronic status and applies these claims expenditure figures to the mix of beneficiaries on EVO by age, gender and chronic status. This translates to a risk-adjusted comparative cost of 16.4% below Emerald expenditures. In other words, EVO claims levels are lower than Emerald claims in nominal terms for the same benefits. Because EVO has a worse risk profile, we would expect EVO claims to be higher than Emerald claims, all else being equal. When we adjust Emerald claims to allow for the EVO beneficiary risk profile, EVO claims levels are even lower, to the value of 17.5%.

These savings arise because of the net effect of:

- A 28.4% lower hospital expenditure,
- A 24.6% lower specialist expenditure out of hospital,
- A 5.4% decrease in GP expenditure out of hospital, and
- A 8.9% decrease in other expenditure.

Annualised savings to GEMS because of the EVO effect amount to R490 million per annum (2018 terms).

### Utilisation and surrogate outcome measures

The savings outlined above emanate from the following observations:

- The hospital admission rate on EVO is 12.0% lower than expected.
- The hospital cost per admission on EVO is 18.7% lower than expected.
- The specialist visit rate on EVO is 19.5% lower than expected.
- The number of GP consultations relative to the number of specialist consultations is 28.3% higher than expected.

## Discussion

The emerging experience on GEMS EVO demonstrates the benefits of care coordination. Visits to GPs are more frequent and there is less doctor hopping. Requiring referrals to access specialist benefits results in lower specialist consultation rates and, as a consequence, fewer hospitalisations.

Emerald Value Option hospital costs are 28.2% lower than what would be expected on the Emerald option for the same risk profile (because of a combination of lower admissions and lower negotiated fees) as depicted in Table 3. Table 4 depicts a similar saving for Q1, 2019. The EVO saved approximately R490m in healthcare costs in 2018. R320m of this can be attributed to hospital expenditure. If applied across the medical schemes industry, it is estimated that EVO care coordination principles could save R20 billion per annum.

**TABLE 3:** Actual and expected Emerald value option expenditure (2018).

Benefit category	Actual spend PLPM	Expected spend PLPM	Variation (%)	*p*
Hospital spend	R439.50	R611.98	−28.2	0.003
Specialist spend, out of hospital	R28.21	R37.42	−24.6	0.070
General practitioner spend, out of hospital	R112.77	R120.59	−6.5	0.388
Other	R777.06	R851.50	−8.7	0.0550
**Total**	**R1357.54**	**R1621.49**	**-16.3**	**0.1356**

*Source*: Government Employees Medical Scheme. Actuarial Report Q1. Pretoria: Government Employees Medical Scheme; 2019^19^

PLPM, Per Life Per Month.

**TABLE 4:** Actual and expected Emerald value option expenditure (Q1 2019).

Benefit category	Actual spend PLPM	Expected spend PLPM	Variation (%)	*p*
Hospital spend	R497.06	R684.01	−27.3	0.001
Specialist spend, out of hospital	R40.04	R51.65	−22.5	0.031
General practitioner spend, out of hospital	R137.31	R140.54	−2.3	0.473
Other	R872.70	R954.13	−8.5	0.003
**Total**	**R1547.12**	**R1830.33**	**-15.5**	**0.283**

*Source*: Government Employees Medical Scheme. Actuarial Report Q2. Pretoria: Government Employees Medical Scheme; 2019^17^

PLPM, Per Life Per Month.

The EVO has also contributed to significantly improved healthcare outcomes by way of reduced unnecessary hospital admissions. The EVO has reduced the hospital admission rate by 11.6% during 2018 as depicted in Table 5. Table 6 further depicts similar reduced hospital admission rate for Q1, 2019. Over 5361 avoidable admissions have been prevented.

**TABLE 5:** Utilisation and surrogate outcome measures (2018).

Variable	Actual	Expected	Variation (%)	*p*
Hospital admissions	23.2%	26.2%	−11.6	0.297
Hospital cost per admission	R25 725	R31 286	−17.8	0.174
Specialist visits per annum	0.51	0.62	−18.5	0.041
General practitioner consultations per specialist consultation	6.67	5.26	26.7	0.315

*Source*: Government Employees Medical Scheme. Actuarial Report Q1. Pretoria: Government Employees Medical Scheme; 2019^19^

**TABLE 6:** Utilisation and surrogate outcome measures (Q1 2019).

Variable	Actual	Expected	Variation (%)	*p*
Hospital admissions	24.3%	27.6%	−12.1	0.282
Hospital cost per admission	R24 589	R29 745	−17.3	0.189
Specialist visits per annum	0.57	0.71	−20.4	0.009
General practitioner consultations per specialist consultation	6.40	4.90	30.8	0.299

*Source*: Government Employees Medical Scheme. Actuarial Report Q2. Pretoria: Government Employees Medical Scheme; 2019^17^

Cost savings are passed on to members in the form of lower contributions. Emerald Value Option members benefit from contributions, which are approximately 15% lower than that of the Emerald option whilst still being able to enjoy the same rich benefits as the Emerald option.

The concept of care coordination is included in the draft National Health Insurance Bill. The draft refers extensively to the championing of primary care and referral pathways. The draft also refers to the procurement of services from contracted providers that meet certain predetermined criteria in terms of the cost and quality of care. The establishment of an efficient network is fully aligned with this principle.

## Conclusion

The study assessed the value proposition of the GEMS EVO, an EDO option requiring special dispensation from the CMS. The findings showed the desired outcomes in terms of cost savings and improved care coordination. A significant decline in health costs of more than 20% was reported; particularly hospital and special costs were noted and this was also statistically significant. Specialist visits also decreased by nearly 20%.

The success of the EVO serves as a blueprint for a progressive benefit design for medical scheme options, which include care coordination based and direct provider contracting.

### Limitations

The study did not conduct a comparison analysis between EVO and EDOs, further analysis in this regard could certainly improve the findings of the study in as far as the performance of EDOs at large. The study did conduct a comprehensive analysis on the set of health quality measures, however, cost and utilization reductions were analysed and reported measures of accessing healthcare at the correct level. The current study reported on surrogate outcome measures, a comprehensive analysis and reporting of other health outcomes and health improvement measures could certainly improve the findings of this study.
